# Setting the research agenda: involving parents in research on children who are HIV‐free

**DOI:** 10.1002/jia2.26150

**Published:** 2023-11-01

**Authors:** Laurette L. Bukasa, Angelina Namiba, Matilda Brown, Estelle Ndu'ngu, Mercy Nangwale, Gillian Letting, Patricia Chirwa, Claire Thorne, Shema Tariq

**Affiliations:** ^1^ UCL Great Ormond Institute of Child Health London UK; ^2^ 4M Network of Mentor Mothers London UK; ^3^ UCL Institute for Global Health London UK; ^4^ Mortimer Market Centre, Central and North West London NHS Foundation Trust London UK

**Keywords:** public engagement, co‐production, women, children, community, HIV

## Abstract

**Introduction:**

There is growing interest in health, developmental and survival outcomes of children who are born HIV‐free to women living with HIV (children born HIV‐free). To date, the research agenda has been largely determined by researchers, funders and policy makers, with limited involvement of parents, who are key stakeholders. Researchers at UCL Great Ormond Street Institute of Child Health in partnership with community‐based organisation 4M Network of Mentor Mothers conducted two workshops with parents in March 2022 to establish research priorities for children born HIV‐free, and key considerations for methodological approaches both to research and engagement with the affected communities.

**Discussion:**

When exploring research on children born HIV‐free, we consider the following: what aspects of current research are aligned with women and parents’ priorities, what is missing and what approaches would be preferred. A holistic approach to research on children born HIV‐free should be prioritised, focussing on a breadth of outcomes and how they intersect. Secondary use of existing data sources should be maximised to facilitate this, with a view of monitoring the long‐term effects of fetal antiretroviral drug exposure alongside other key health and developmental outcomes. Involving and engaging with parents, and children where possible, must be at the heart of research design to maximise relevance and impact of findings for the affected communities. Potential barriers to engaging with individuals who were children born HIV‐free include parental disclosure and individuals not identifying as a child born HIV‐free to a mother living with HIV. Stigma‐free language must be incorporated into the vocabulary of researchers and other stakeholders, avoiding reference to exposure; we propose the term “children born HIV‐free.”

**Conclusions:**

Mothers and parents living with HIV should be involved in research about their children born HIV‐free and are key in identifying research priorities so that findings may translate into an impact on their children's health and wellbeing. Meaningful involvement of women living with HIV through trusted community partners is an effective mechanism by which to elicit views on research about their children.

## 1 INTRODUCTION

Successes in preventing vertical transmission (VT) have led to substantially fewer children being born with HIV [1]. In parallel, the number of children who are born HIV‐free to women living with HIV* (*children born HIV‐free will henceforth refer to children born to women living with HIV who remain HIV‐free throughout childhood) has increased to an estimated 15 million globally, with more than half living in just four sub‐Saharan African countries [2]. In the UK, there are approximately 900 pregnancies in women living with HIV each year with a VT rate <0.4% since 2012 among diagnosed women [3].

The first 1000 days of life (conception to approximately 24 months) is a critical period of development for children, where factors relating to the maternal and external environment can play an important role in modifying life‐long risk of health and developmental outcomes [4]. Children born HIV‐free develop in an intrauterine environment where HIV, antiretroviral drugs and other factors, such as anxiety and depression, affect the mother in pregnancy [5]. Growing evidence from predominantly low‐ and middle‐income settings suggests that children born HIV‐free may have sub‐optimal health and survival outcomes compared with children born to women without HIV [6, 7, 8, 9]. Specific antiretrovirals [10, 11], HIV viral factors [12, 13] and adverse birth outcomes, such as preterm birth [14], may contribute to these divergent outcomes, but many questions remain.

The multilevel response to improve outcomes for children born HIV‐free have included interventions to reduce mortality, support programmatic scale‐up and effectiveness of prevention of VT programmes, and minimise health and developmental disparities within a framework where children are alive, HIV‐free and thriving [15]. With progress being made to improve survival and reduce VT rates globally [16], the research landscape is increasingly focussed on children thriving. Recent findings have demonstrated that in addition to existing evidence on increased morbidity, developmental delay may disproportionately affect children born HIV‐free in sub‐Saharan Africa [17, 18]. This could involve pathways relating to indirect effects of maternal HIV, such as adverse maternal mental health, that is depression and stress [17, 19] and possible direct effects of exposure to maternal HIV, co‐infections and Antiretroviral therapy (ART) in utero [20], although no data exist for the UK.

There is growing recognition of the importance of including pregnant people and parents living with HIV at all stages of research, beyond being research participants, in accordance with equitable research principles and UNAIDS policy for greater involvement of people living with HIV [21, 22]. Successful examples of co‐production in research and policymaking exist; such as the inclusion of mothers living with HIV in the study team and the advisory board of the NOURISH‐UK study on HIV and infant feeding [23] and community‐led WHO consultation with women living with HIV when preparing updates to guidelines [24]. However, there are areas of research where women living with HIV are excluded from participating and shaping research. For example, pregnant and breastfeeding women have been routinely excluded from clinical research on antiretroviral drugs, perpetuating the widening evidence gap on drug safety during this period [25]; when enabled to contribute to discussions on this topic, women had strong views that challenged long‐standing protocols and advocated for them to make informed choices about participation [26]. Communities can have differing views on research to researchers, yet parents living with HIV are rarely involved in shaping research on children born HIV‐free, despite often being the main caregivers with key insights into their children's health. Even less common is the involvement of children born HIV‐free themselves, although models for meaningful involvement of children and young adults living with HIV exist [27].

Researchers at UCL conducting research on children born HIV‐free in the UK and 4M Network of Mentor Mothers convened two workshops in March 2022 with six biological mothers of children born HIV‐free from Black ethnic backgrounds to explore components for a research agenda in the UK and globally. Building on the outcomes of these discussions, we provide key learnings for stakeholders involved in research on children born HIV‐free.

## 2 DISCUSSION

**Figure 1:**
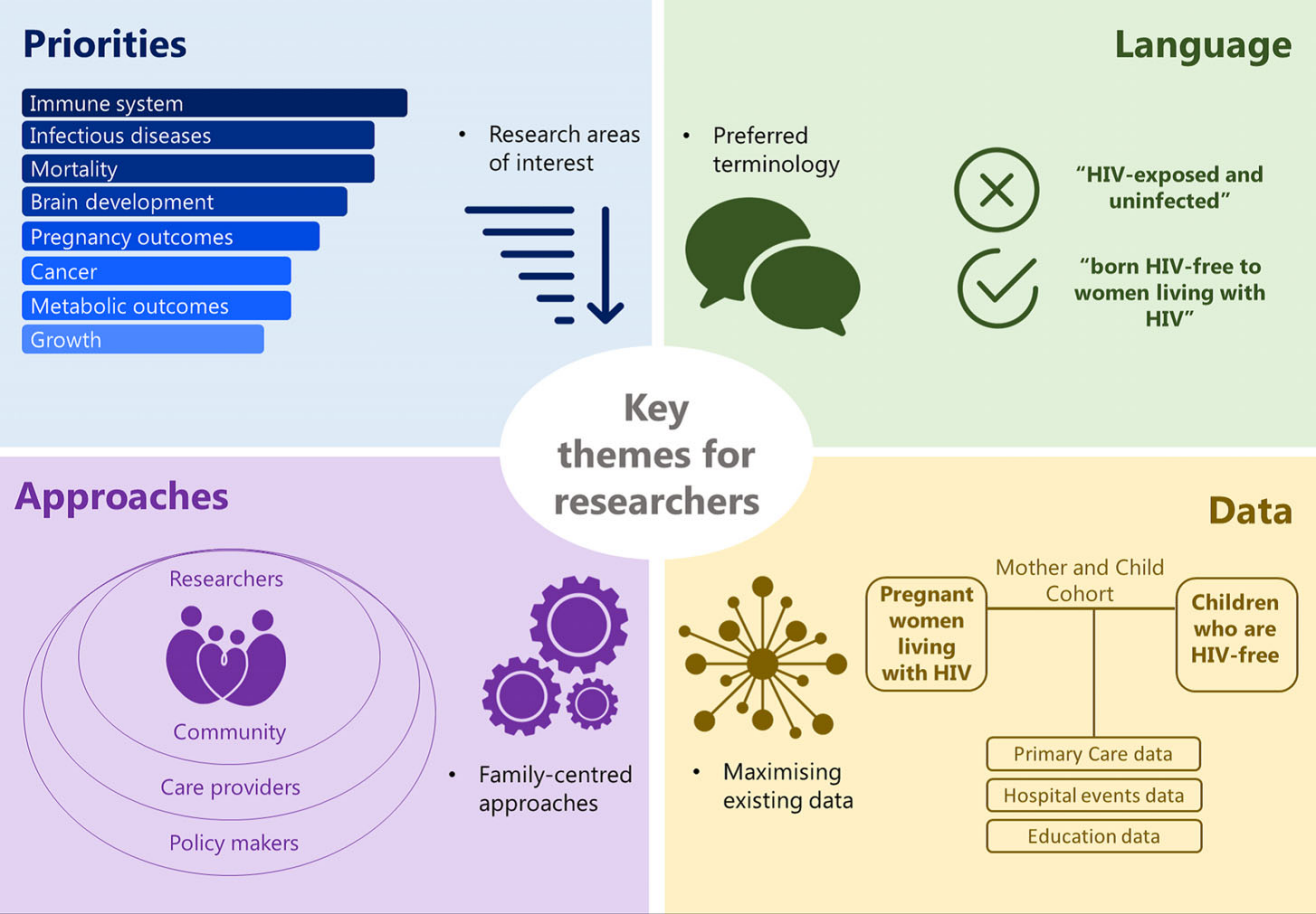
Key themes for research on children born HIV‐free. Top left corner shows the group priority research areas, top right corner illustrates terminology preferences, bottom left corner illustrates a family‐centred approach to research and engagement with other stakeholders and bottom right corner shows a data infrastructure to explore outcomes.

In our workshops, six parents living with HIV established themes for research on children born HIV‐free, including priorities, an infrastructure for utilising existing data source, language preferences and approaches (Figure 1).

### 2.1 Outcomes of interest

There has been long‐standing recognition of the need to monitor the longer‐term health and development of children born HIV‐free in the UK, in the context of routine discharge from clinical care after confirmation of HIV‐free status at approximately 24 months of age [28, 29]. The primary concern for families affected by HIV in the UK was the potential adverse effects associated with early life exposures to antiretrovirals, and there was explicit support for researchers to address the significant knowledge gaps that still exist. In view of this, parents expressed a preference for a wide range of outcomes to be investigated, regardless of perceived severity. For example, dental conditions may not be considered a severe adverse outcome by researchers, yet parents are concerned about such conditions as they are common and impact their children's wellbeing. An exploration of the long‐term health effects or benefits of breastfeeding was also identified as a key outcome; breast/chestfeeding is less common in high‐income settings like the UK, but increasingly becoming the infant feeding option of choice [30, 31]. Limited data on the effects of neonatal HIV post‐exposure prophylaxis (PEP) [32] should also be addressed. This is particularly salient given the increasing prevalence of breast/chestfeeding among people with HIV [33], prolonging exposure to ART through human milk and infant PEP for the duration of breast/chestfeeding in some settings [34].

Sub‐optimal immune system development and increased risk and severity of infectious diseases have been reported in cohorts of children born HIV‐free both in high‐income and low‐income settings, although not specifically in the UK [35, 36]. Parents observed that their children born HIV‐free may experience infections more often than other children, with particular mention of tonsilitis. Increased frequency and severity of infections in children born HIV‐free may have multidimensional impacts beyond health, such as poor school attendance which can lead to social service involvement. This highlights the potential upstream effects of poorer health in childhood on later life, mediated by educational attainment. Engaging parents can yield an understanding of the individual‐level impact of findings on infections and immune system development of children born HIV‐free.

### 2.2 Utilising existing data and pioneering data linkages

When considering data collection methods, secondary use of administrative data (information generated when interacting with public services [37]) was desirable for several reasons, including efficiency, cost, and robust ethical and data governance processes. A key advantage among parents was the potential for linkage using multiple datasets that could facilitate a broader understanding of health and developmental outcomes among children born HIV‐free. For example, there is conflicting evidence regarding neurodevelopmental outcomes in children born HIV‐free in low‐ and middle‐income countries [20, 38] but there are no data for the UK context at present. Linking health and educational records would enable an investigation of possible disparities in educational attainment and explore potential drivers. Similarly, primary care data could be valuable for understanding health from pregnancy through to childhood as the main entry point to clinical care pathways, containing information on morbidity (less severe than hospital events), prescriptions and vaccinations.

Utilising existing data sources to explore health and developmental outcomes among children born HIV‐free minimises the burden on the affected communities to contribute to research as participants. Currently, much of the data from this population come from prospective observational cohort studies that require follow‐up visits and are associated with costs in both time and money to participants and their families. In addition, the perceived burden of follow‐up may act as a barrier to recruitment, threatening the feasibility of studies [39] that are already limited in their capacity to address long‐term outcomes with respect to research funding. However, these benefits for secondary use of existing data should be considered alongside its limitations including limited scope to explore beyond what has already been collected.

### 2.3 Language and terminology

The use of language for people living with HIV has evolved, with greater recognition of its power to perpetuate stigma and discrimination [40]. There is now a commitment among many key stakeholders, including researchers and clinicians, to adopt terms that are destigmatising and people‐centred, illustrated by the People First Charter [41]. Parent(s), who have experienced this evolution first‐hand, place great importance on getting the language right from the outset for children born HIV‐free. Terms such as “uninfected” can be viewed as clinical and stigmatising, and the use of acronyms such as “CHEU” (children who are HIV‐exposed but uninfected) dehumanising when engaging in dialogue about children born HIV‐free. There was a preference for terms such as “children born without HIV,” “children born HIV‐free” or “children who are HIV‐free” among parents, and while current terminology used among stakeholders (children who are HIV‐exposed and uninfected) adhere to the People First Charter [42], greater consideration should be given by researchers to how they refer to this population.

### 2.4 A family‐centred approach for setting the research agenda

#### 2.4.1 Women and pregnancy

Exploring outcomes of children born HIV‐free within the context of the woman and mother could offer an alternative perspective on inequalities within and between populations:

“*For a healthy pregnancy, birth and child, a woman must be physically and mentally well*.”

Maternal mental health in the context of HIV in pregnancy is an underdeveloped area of research in the UK. Women living with HIV are disproportionately affected by mental health difficulties [43, 44], driven in part by social disadvantage [44, 45]. Investigating the impact of maternal mental health on pregnancy and both short‐ and long‐term child outcomes may elucidate pathways that may be driving health and developmental inequalities and reveal opportunities for potential interventions in similar settings where evidence of developmental differences have been observed [46]. The persistence of adverse parental mental health can affect children born HIV‐free directly and indirectly; direct effects include children being more likely to be absent from school or display behavioural problems [47], and indirect effects could include the risk of HIV‐related death among parents experiencing mental health difficulties [48], which could, in turn, increase the risk of adverse childhood experiences (i.e. parental bereavement) among children born HIV‐free, analogous to their counterparts living with HIV [49, 50].

#### 2.4.2 Engaging children born HIV‐free

Parents identified children born HIV‐free as experts‐by‐experience who should be involved in research. However, engaging children born HIV‐free is associated with several challenges.

Firstly, parents need to talk about their HIV status to their children, but rates of parental disclosure are low globally [51, 52]. While there are benefits of this sharing for the parent‐child relationship [53], it is conditional upon an acceptance of the parent's HIV status themselves, which could involve overcoming self‐stigma, cultural and family‐related factors. Even if a child or young adult is aware of their parent(s)’ HIV status, opportunities to involve them in discussions about research are limited. There is a lack of community‐based organisations for this population in the UK and beyond, and the absence of routine population‐level clinical follow‐up of children born HIV eliminates the possibility of approaching them opportunistically. However, children and young adults can be identified through their family members living with HIV; for example, HIV‐free siblings of young people with perinatally acquired HIV were recruited as controls in the UK's AALPHI study [54].

Furthermore, those who are HIV‐free and aware of their HIV‐free status may not perceive themselves as different from children who were born to women without HIV. Engaging a population that do not consider themselves as at risk of adverse outcomes or do not identify as “HIV‐exposed” is a significant barrier to the co‐production of research on children born HIV‐free. A driving factor for children and young adult's dissociation from being “HIV‐free” could be related to HIV‐associated stigma and fear of bullying if their parent(s)’ status became common knowledge. Researchers must also consider the capacity for children and young adults to engage with and become involved with research. Some will also be navigating social and economic disadvantage which can be prevalent in communities affected by HIV [55, 56], as well as potential illness within their families.

There are likely to be varying perspectives on being “HIV‐free” among children and young adults born to women living with HIV, highlighting the importance of involving them in discussions around the research agenda from inception, as their views on research and its relevance to their lives may differ to their parents [57].

### 2.5 Maximising research impact

Research on children born HIV‐free can be poorly disseminated to communities which minimises impact. When considering stakeholder groups for dissemination of research findings, children and/or young adults born HIV‐free should be prioritised along with their parent(s) living with HIV. Children born HIV‐free should be given access to information on research at an appropriate age of comprehension [58] using specific content created for them, and efforts made to engage within settings in which they feel comfortable, that is podcasts, social media or attending youth events. Parents living with HIV should be disseminating findings alongside researchers as self‐advocates, particularly in health and social care settings. Dissemination strategies must be co‐produced with children born HIV‐free and their parents to target appropriate key stakeholders, and to tailor the communication of findings to each audience.

## 3 CONCLUSIONS

In this commentary, we draw upon findings from workshops with six parents living with HIV who provided feedback on priorities and approaches for research on children born HIV‐free. We have highlighted how parents and children affected by HIV have unique and important insights into research on children born HIV‐free. It is vital that academics, clinicians, funders and other stakeholders centre their voices to increase relevancy and maximise the impact of research. In collaboration with parents living with HIV, we have identified a set of research priorities and recommendations for the research agenda that are based on selecting a broad range of outcomes; using and linking existing data; using stigma‐free language; and employing a holistic, life‐course approach that values involving parents and children. We propose an extension to existing frameworks to decolonise global health that foreground the need for equitable research partnerships [59], by including this often marginalised population of parents living with HIV, and their children, as research partners in setting the research agenda in this growing population of children. Identifying potential barriers for involving affected communities is a necessary component in achieving this, and exploration of these challenges can facilitate ongoing dialogue with communities on how to overcome them.

## COMPETING INTERESTS

CT has received funding from ViiV Healthcare via the PENTA Foundation for a pharmacovigilance study on drug use and safety in pregnancy. ST has previously received speaker honoraria and funding for the preparation of educational materials from Gilead Sciences.

## AUTHORS’ CONTRIBUTIONS

LLB and AN conceived concept of the project. LLB, AN, CT and ST designed workshop. AN, MN, PC, GL, MB and EN contributed to workshop content. LLB wrote the manuscript. AN, CT and ST reviewed the manuscript.

## Data Availability

Data sharing not applicable to this article as no datasets were generated or analysed during the current study.
